# Uncoupling of VEGF with Endothelial NO as a Potential Mechanism for Abnormal Angiogenesis in the Diabetic Nephropathy

**DOI:** 10.1155/2013/184539

**Published:** 2013-12-09

**Authors:** Takahiko Nakagawa, Waichi Sato, Tomoki Kosugi, Richard J. Johnson

**Affiliations:** ^1^TMK Project, Kyoto University Graduate School of Medicine, Kyoto 606-8397, Japan; ^2^Department of Nephrology, Nagoya University Graduate School of Medicine, 466-8550, Japan; ^3^Division of Renal Diseases and Hypertension, University of Colorado Denver, Aurora, CO 80045, USA

## Abstract

Abnormal angiogenesis is a well characterized complication in diabetic retinopathy and is now recognized as a feature of diabetic nephropathy. The primary growth factor driving the increased angiogenesis in diabetic retinopathy and nephropathy is vascular endothelial growth factor (VEGF). While VEGF is considered an important growth factor for maintaining glomerular capillary integrity and function, increased action of VEGF in diabetic renal disease may carry adverse consequences. Studies by our group suggest that the effects of VEGF are amplified in the setting of endothelial dysfunction and low nitric oxide (NO) levels, which are a common feature in the diabetic state. The lack of NO may amplify the effects of VEGF to induce inflammation (via effects on the macrophage) and may lead to dysregulation of the vasculature, exacerbating features of diabetic renal disease. In this review, we summarize how an “uncoupling” of the VEGF-NO axis may contribute to the pathology of the diabetic kidney.

## 1. Abnormal Angiogenesis Is a Characteristic Feature of Diabetic Nephropathy

The first description documenting abnormal angiogenesis in the diabetic kidney is from a 1987 study by Østerby and Nyberg [1]. These authors reported that patients with long-term type 1 diabetes showed an increase in capillaries in the renal biopsy that were both within and surrounding the glomeruli. Other investigators later demonstrated similar findings in type 2 diabetic patients with kidney disease [2, 3]. In these patients, 1–5% of glomerular capillaries were found to contain aberrant vessels. Interestingly, the abnormal vessels were also present in Bowman's capsule or in the glomerular vascular pole, presenting as an “extra efferent arteriole” [1, 4]. A Japanese research group examined human kidney samples from 94 patients with diabetes and performed detailed analyses of serial sections using computer-generated three dimensional images [5]. They reported that the abnormal vessels were often found to be anastomosed to the lobular structure of the intraglomerular capillary network, mainly to afferent branches through the widened vascular hilus, while the distal end of the vessels was connected to the peritubular capillary. Morphologically the endothelial cells were often swollen early in the disease only to become shrunken as diabetes progressed [6, 7]. Another interesting finding was that the aberrant proliferation of blood vessels was not infrequent in diabetic patients even during the first two years of disease [5], indicating that the development of these vessels could occur in the early phases of diabetic nephropathy.

Similar to human diabetic kidney disease, some diabetic animal models also developed excessive numbers of capillary vessels. For instance, Nyengaard and Rasch identified abnormal glomerular capillaries in an animal rat model induced by streptozotocin [8]. The db/db mice also exhibit an increase in endothelial cell number and an elongation of capillaries in their glomeruli [9, 10]. However, it should be noted that in the later stages of diabetic nephropathy, there is often a loss of capillaries in both human and animal models [2, 11, 12]. A decrease in VEGF expression in advanced stage of diabetic nephropathy could account for such capillary loss [2, 11, 12].

## 2. VEGF Is Deleterious in Diabetic Kidney as Opposed to Nondiabetic Renal Disease

VEGF is a critical growth factor for endothelial cells, especially in the kidney. Podocytes and proximal tubular epithelial cells are likely major sources for VEGF which binds to receptors on the glomerular and peritubular endothelial cells, respectively. Under conditions in which local VEGF levels fall acutely, a loss of capillaries occurs, leading to lesions that may appear similar to a thrombotic microangiopathy. In progressive nondiabetic kidney disease, a loss of VEGF may occur more slowly, leading to a loss of capillaries in association with reduced renal function and fibrosis. Under these cases, the administration of VEGF can stimulate capillary growth and improve the kidney lesions [13–15]. Given these facts, VEGF seems to be indispensable for renal normal physiology and a loss of VEGF may play an important role in both acute and chronic kidney diseases.

In contrast, an excessive amount of VEGF is likely a contributory factor for diabetic kidney disease. This nature was first shown in a 1999 study, in which an increase in renal VEGF/VEGFR2 expression was observed in streptozotocin (STZ) induced diabetic rat [16]. Likewise, we also documented an increase in glomerular VEGF expression, which was associated with diabetic glomerular injury in the diabetic eNOSKO mice [17]. These findings were confirmed in human diabetic nephropathy, in which VEGF was found to be increased in both renal biopsies and urine [3, 18].

To determine its role in diabetic kidney disease, several investigators have attempted to inhibit the excessive VEGF. For instance, anti-VEGF antibody was the first to be tested while a pharmacological inhibitor was also used in the several types of diabetic rodents, including STZ induced diabetic rats, db/db mice, and Zucker rats [19, 20]. In general, blocking VEGF consistently demonstrated protective effects, such as a reduction in urine albumin excretion, an inhibition in glomerular matrix expansion, and podocyte protection. Likewise, Ku and colleagues utilized a molecular technology to overexpress sFlt-1 (a soluble VEGFR1) in podocytes to locally block VEGF function in STZ diabetic mice. This treatment had similar beneficial effects as systemic VEGF inhibitors [21]. While these studies unfortunately did not examine the direct effect of such therapies on the development of abnormal angiogenesis, they do provide supporting evidence that excessive VEGF expression may contribute to diabetic nephropathy.

## 3. Why Is VEGF Deleterious in Diabetic Nephropathy? 

While VEGF is capable of producing several biological factors, one of the most important factors could be the endothelial nitric oxide (NO) because endothelial NO was found to potently protect the vasculature in several ways, including stimulating vascular relaxation and having both anticoagulation and anti-inflammatory effects. As such, it is likely that the vascular protections of VEGF might be via stimulating NO production. In contrast, a lack of NO could turn VEGF to be deleterious in vascular system. Zhao et al. reported that blocking NO production induced vascular remodeling and inflammation along with upregulation of VEGF. Importantly, blocking VEGF action resulted in ameliorating such injury, indicating that VEGF could be deleterious in vascular system in the absence of NO [22]. Thus, endothelial NO could be a key factor to regulate VEGF function.

**Figure 1:**
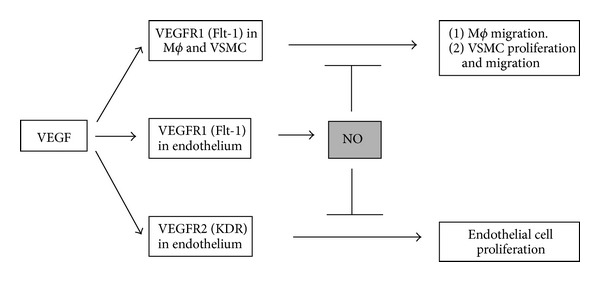
Central role of NO in regulating VEGF system in endothelial cells. In endothelium, VEGFR1 contributes to NO production whereas endothelial cell proliferation is regulated by VEGFR2. VEGFR1 is also expressed in macrophage as well as VSMC. In the normal setting, endothelial cells produce NO, which negatively regulates endothelial cell proliferation, macrophage migration, and VSMC activation to maintain the well balanced vascular integrity. In contrast, endothelial NO bioavailability is reduced in certain physiological conditions, such as diabetes. In the case that NO bioavailability is reduced in endothelium, a compensatory increase in VEGF expression as well as a disruption of negative regulation in vascular system in response to VEGF occurs. As a consequence, VEGF engages VEGFR2 to enhance endothelial cell proliferation while VEGFR1 on macrophage and VSMC can be activated to induce vascular injury.

How can NO regulate VEGF action? In 2001, Dunk and Ahmed addressed this issue with the tumor epithelial cells. They concluded that cell proliferation is mediated by VEGFR2 while VEGFR1 stimulation resulted in NO production, suggesting that these two actions are independently regulated by two different receptors. They also found that NO, which was mediated by VEGFR1 stimulation, could negatively regulate VEGFR2-mediated mitogenesis [23]. Given these facts, we hypothesized that the combination of increased VEGF with an impaired endothelial NO response might play a role in the abnormal angiogenesis observed in diabetes (Figure 1).

## 4. Endothelial NO Availability Is Reduced in Diabetic Condition

Is NO bioavailability reduced in the diabetic kidney? In this regard, many studies have documented that diabetes is associated with a reduction in NO bioavailability. The underlying mechanisms appear to be diverse, but many diabetes-related factors are likely involved, including hyperglycemia [24], advanced glycation end-products [25], uric acid [26], ADMA [27] and oxidative stress [28], and are actually able to reduce NO bioavailability.

Gene polymorphisms in endothelial nitric oxide synthase (eNOS) may be also a factor, which is involved in regulating NO levels [29] because eNOS is the principal enzyme producing NO in endothelial cells. Based on such assumption, eNOS gene polymorphisms have been examined in diabetic patients by several investigators. However, the role of such genetic alteration remains unclear as some [30–33], but not all, studies [34–37] documented a positive association of specific eNOS polymorphisms with diabetic nephropathy.

Alternatively, Hohenstein et al. performed immunohistochemistry to investigate eNOS expression in type 2 diabetic patients and found that eNOS expression was increased in glomeruli in patients with diabetes [38]. Similarly, STZ induced diabetic rats were found to exhibit an increase in eNOS expression in endothelial cells in both the afferent arterioles and the glomerulus [39]. While these lines of evidences do not meet our assumption, we should be aware that eNOS expression is not always correlated with its activity. In general, the production of NO requires eNOS to be “coupled” while, in turn, “uncoupled” eNOS generates superoxide as opposed to NO. Brodsky et al. found that high glucose induces uncoupling of eNOS, which causes a reduction in NO bioavailability and an increase in superoxide production [40]. Likewise, Komers et al. found that eNOS in diabetic kidney may also exist in the uncoupled form where it localized to the cytosolic fraction. Since eNOS activation also requires the translocation into plasma membrane in the coupled form [41], it is likely that the upregulated eNOS in diabetes might be an inactivated form.

## 5. Uncoupling of VEGF with Endothelial NO Causes Abnormal Angiogenesis in the Diabetic Kidney

Regarding such notion, our first insight came from studying STZ induced diabetes in the mouse lacking eNOS (eNOSKO) [12]. VEGF expression was increased in both diabetic wild type and diabetic eNOSKO mice. Since eNOS was genetically deleted, the kidney exhibited the condition of being upregulated. However, diabetic eNOSKO mice developed much more severe clinical manifestations that resembled overt diabetic nephropathy in humans. For example, this mouse was found to develop hypertension, massive albuminuria, and renal dysfunction [12]. This mouse model also exhibited higher mortality rates from progressive renal disease [12, 42]. Histological manifestations of diabetic eNOSKO mice also mimic those of human diabetic nephropathy. In fact, this mouse model developed not only the early manifestations, such as mesangial expansion and glomerular basement membrane thickening, but also advanced lesions including mesangiolysis, Kimmelstiel-Wilson-like nodules, arteriolar hyalinosis, and tubulointerstitial disease [12].

Importantly this mouse model demonstrated that excessive numbers of small blood vessels were induced around glomeruli where normal vessels do not normally exist. Interestingly, this is the same location where abnormal blood vessels are observed in human diabetic kidney disease [12]. In addition, an increase in endothelial cell number in both glomerular and peritubular lesions was found to exhibit a proliferative response [12], which could be a potential mechanism for the development of abnormal angiogenesis in this mouse model. These studies suggested that the combination of high VEGF and low endothelial NO levels might be responsible for the abnormal enhanced endothelial proliferation in this mouse model.

We next used the cell culture system to test our hypothesis. Here we evaluated whether a lack of NO could alter the proliferative effects of VEGF on endothelial cells [43]. Our primary finding was that blocking NO using either an NO synthase inhibitor or high glucose condition could enhance the proliferative response of endothelial cells to VEGF [43]. Next issue was to investigate the mechanism. VEGF is known to bind two different receptors, raising the question of which one might be more important in mediating these effects. Our study demonstrated that VEGFR2 was primarily responsible for the cell proliferative response in endothelial cells [43]. However, VEGFR1 was the primary receptor responsible for stimulating NO since a VEGFR2 inhibitor failed to block NO production as well as eNOS phosphorylation in response to VEGF in endothelial cells[43]. Such findings are consistent with a 2001 study demonstrating that distinct role between VEGFR1 and VEGFR2 in nonendothelial cells [23]. Taken together, VEGF likely acts on endothelial cells by two pathways; one elicits endothelial proliferation through VEGFR2 while the other the activating VEGFR1 for induction of NO, which negatively regulates the VEGFR2-mediated proliferative response.

Nonetheless, endothelial cells might not be the only target for this uncoupling condition. In our animal model, we found that an increase in macrophage infiltration was observed in glomeruli where VEGF expression was upregulated in diabetic eNOSKO mice [17], suggesting that the uncoupling condition could also mediate macrophage migration. In this regard, NO administration could fix such VEGF-NO balance, resulting in preventing macrophage migration if our assumption is correct. In cultured macrophage cell line, we found that VEGF was able to induce macrophage migration in Boyden chamber assay while administration of NO donor alleviated such migration in response to VEGF [17]. Hence, such uncoupling theory could be also applied to macrophage infiltration in the diabetic nephropathy.

We also generated a mouse model in which we were able to test the role of NO in the VEGFR2-mediated endothelial proliferative response [44]. In this experiment, we utilized the adenoassociated virus (AAV) to overexpress a VEGF mutant (mtVEGF), which could only bind to VEGFR2. Following the injection, mice underwent uninephrectomy to amplify any renal lesions. Wild type (WT) mice were also treated in the same way as a control group. We also performed the study using mice lacking eNOS (eNOSKO) to further allow us to specifically analyze the relationship between VEGFR2 signal and endothelial NO in the kidney. Overexpression of mtVEGF resulted in increased angiogenesis and lowering of blood pressure in both types of mice whereas such effects were greater in eNOSKO than WT [44]. In addition, mtVEGF-AAV also caused severe mesangial injury with increased proliferation associated with elevated PDGF, PDGF-*β* receptor, and VEGFR2 in eNOSKO mice compared to similarly treated WT mice [44]. These data indicate that enhancing VEGFR2 signal could induce aberrant angiogenesis which could be further exaggerated in the absence of eNOS in the kidney.

## 6. Can a Low in VEGF Expression Also Be Deleterious Even in Diabetic Nephropathy?

Our studies clearly demonstrate that an elevation in VEGF may result in deleterious consequences in diabetic nephropathy, primarily by overactivation of the VEGFR2 pathway in the setting of endothelial dysfunction. However, as diabetic nephropathy continues, there may actually be a reverse situation where VEGF expression falls in association with chronic glomerular and tubular injury. For example, there are a couple of studies showing that VEGF expression is reduced in human diabetic nephropathy within sclerotic areas and in nodular lesions in the glomeruli [45, 46]. Baelde et al. documented a 2.5-fold reduction in VEGF expression late stage diabetic nephropathy in association with a loss of endothelial cells and a reduction in podocytes [11]. Such interesting concept was highlighted in an elegant study by Hohenstein and colleagues in which VEGF activity was increased only in the mildly injured glomeruli but significantly decreased in more severely injured glomeruli [2]. Given these facts, a low level of VEGF is also undesirable in diabetes and is likely to manifest as the kidney disease progresses.

Why could a lowering VEGF be also deleterious in the diabetic nephropathy? It could be because normal kidney requires a certain level of VEGF to maintain integrity of renal function. In general, normal kidney is composed of abundant vessels so that a physiological level of VEGF is required to maintain such vascular system. Perhaps, diabetic kidney does so as well. In fact, we previously demonstrated that blocking VEGF rather deteriorated tubulointerstitial injury. Importantly such injury was accompanied with a loss of peritubular capillary, indicating that blocking VEGF made its level too low so that peritubular capillary system could not be maintained. Therefore a loss of peritubular capillary seems to be a cause for deterioration of tubulointerstitial injury. Recently, this notion was tested by other researchers by using conditional mouse model. Sivaskandarajah et al. used an inducible Cre-loxP gene-targeting system that enabled genetic deletion of VEGF-A selectively from glomerular podocytes of wild type mice, and then type 1 diabetes was induced in mice using streptozotocin (STZ). Importantly, this system allowed them to reduce VEGF level which was lower than physiological level. As a consequence, a deletion of VEGF resulted in more severe kidney injury in diabetes [47]. Hence, an important message could be that if VEGF is either too low or too high, this factor could be deleterious. Rather, maintaining VEGF at physical level could be protective in the diabetic kidney.

## 7. How Could the Uncoupling Be Fixed?

First of all, we tested the effect of insulin therapy in this model [12]. Controlling blood sugar was found to alleviate the upregulation of VEGF and prevent the progression of diabetic glomerular injury, suggesting that such beneficial effect of insulin could be due in part to a fixation in VEGF-NO balance.

We next examined the effect of the renin-angiotensin system (RAS) blocking [48]. In contrast to diabetic wild type mice, RAS blockades failed to slow the progression of kidney injury in the diabetic eNOSKO mice. Unfortunately, we did not address the issue of VEGF-NO balance in that study. Nevertheless, similar refractoriness to ACEI/ARB in diabetic nephropathy was also reported by other investigators [49, 50]. Common nature of these three studies was that renal injury was relatively severe in the animal models. While a clear mechanism for refractoriness to ACEI/ARB remains undetermined, ACEI/ARB may be no longer protective once diabetic nephropathy progresses severely.

Finally, next compound we used was nicorandil, which has two pharmacological actions; one is to donate NO while the other action is to open K-channel dependently on ATP [51]. We expected that by donating NO, nicorandil could fix the balance of VEGF with NO. As expected, nicorandil was found to exhibit some protection as evidenced by a reduction in urinary protein excretion and slowing of the progression of glomerular injury in accompany with an increase in urinary NOx level [51]. These findings suggest that an NO donor, nicorandil, could correct the balance of VEGF with NO and be a therapeutic option to treat diabetic nephropathy in case that kidney would be under the uncoupling condition.

## 8. Translation of Animal Study into Clinical Medicine

It must be still immature and too preliminary to translate this concept to clinical medicine. However, it might not be impossible to apply this concept in the future study. In such case, the first step could be examining renal VEGF and endothelial function. Urinary VEGF could be a marker of VEGF production in the kidney as its increase in urine was found to be positively associated with a degree of urinary albumin excretion in patients with type 1 diabetes [18]. In turn, endothelial function can be evaluated by several measures which have been already established in clinical medicine. For instance, urinary level of NOx could be a good marker of endothelial dysfunction as it was found to be reduced in type 2 diabetic patients with microalbuminuria compared to those with normoalbuminuria [52]. Plasma von Willebrand factor could be also used as a good marker for endothelial function in diabetic patients with proteinuria [53–55]. Alternatively, the flow mediated vasodilatation or acetylcholine-induced vasodilatation can be also clinically used to evaluate endothelial function. Nevertheless, if any measures find the combination of high VEGF with endothelial dysfunction, such patients might be under the uncoupling condition. In such cases, either donating NO or cautiously blocking of VEGF could be considered as a therapeutic option.

## 9. Conclusions

**Figure 2:**
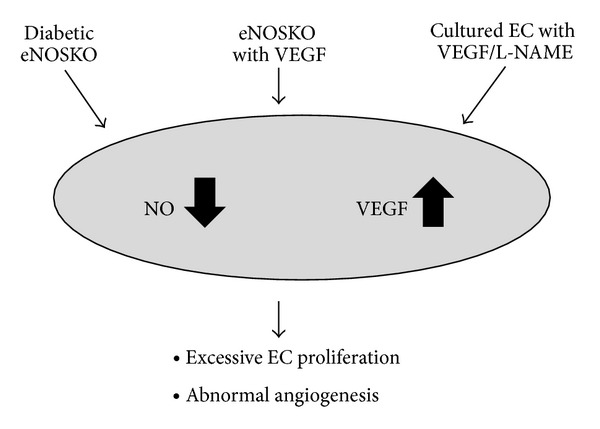
Uncoupling of VEGF with NO could be a pathway to induce abnormal angiogenesis. Three distinct conditions commonly cause uncoupling condition, leading to abnormal angiogenesis.

In summary, physiological levels of VEGF are required for the maintenance of normal renal architecture and function. In case of diabetes, VEGF expression is induced and could exhibit some deleterious effects. In particular when upregulation of VEGF is coupled with endothelial dysfunction, such combination can have a role in driving diabetic renal disease (Figure 2). High levels of VEGF may have a role in abnormal angiogenesis, macrophage activation, and even mesangial expansion. In contrast, as disease progresses, VEGF levels may fall, resulting in endothelial cell loss and capillary rarefaction.
